# Validation of an LC–HRMS Method for Quantifying Indoxyl Sulfate and *p*-Cresyl Sulfate in Human Serum

**DOI:** 10.3390/molecules30040782

**Published:** 2025-02-08

**Authors:** María Rodríguez-García, Irene Martínez, Irene Aliart, Jaime I. Sainz de Medrano, Nayra Rico, Víctor Joaquín Escudero-Saiz, Francisco Maduell, Manuel Morales-Ruiz, Gregori Casals

**Affiliations:** 1 Biochemistry and Molecular Genetics Department, CDB, Hospital Clínic of Barcelona, 08036 Barcelona, Spain; mrodriguezg@clinic.cat (M.R.-G.); imartin1@clinic.cat (I.M.); ialiart@clinic.cat (I.A.); jisanz@clinic.cat (J.I.S.d.M.); nrico@clinic.cat (N.R.); 2 Nephrology and Renal Transplantation, Hospital Clínic of Barcelona, 08036 Barcelona, Spain; vjescudero@clinic.cat (V.J.E.-S.); fmaduell@clinic.cat (F.M.); 3 Institut d’Investigacions Biomèdiques August Pi i Sunyer (IDIBAPS), 08036 Barcelons, Spain; 4 Centro de Investigación Biomédica en Red de Enfermedades Hepáticas y Digestivas (CIBERehd), 28029 Madrid, Spain; 5 Department of Biomedicine, Faculty of Medicine and Health Science, University of Barcelona, 08036 Barcelona, Spain; 6 Department of Fundamental and Clinical Nursing, Faculty of Nursing, University of Barcelona, L’Hospitalet de Llobregat, 08907 Barcelona, Spain

**Keywords:** chronic kidney disease, uremic toxins, high-resolution mass spectrometry, micro-liquid chromatography, hemodiafiltration, metabolomics

## Abstract

Accurate quantification of indoxyl sulfate (IndS) and *p*-cresyl sulfate (pCS) is essential for understanding their role in chronic kidney disease (CKD) progression and for developing strategies to mitigate their harmful effects, including cardiovascular morbidity and renal fibrosis. Advances in liquid chromatography–high-resolution mass spectrometry (LC–HRMS) enable the integration of powerful diagnostic tools into clinical laboratories. Along with accurate quantification, precise mass measurements allow for untargeted compound identification. Methods. An LC–HRMS was validated for quantifying IndS and pCS in human serum, following EMA guidelines. The method involved protein precipitation with methanol, micro-LC for chromatographic separation, and detection based on accurate mass, with simultaneous high-resolution full-scan acquisition. Clinical samples from patients with varying degrees of renal insufficiency and samples obtained before and after hemodiafiltration were analyzed. Results. The method demonstrated acceptable linearity, precision, and accuracy. The measurement range for both analytes was from 100 to 40,000 ng/mL. Serum levels of IndS and pCS correlated with decreased renal function. After hemodiafiltration, there was a significant reduction of IndS (50%) and pCS (43%). Simultaneous untargeted analysis allowed to identify metabolites significantly underexpressed after hemodiafiltration. Conclusions. An accurate LC–HRMS method was validated for the quantification of IndS and pCS serum levels in patients with CKD, providing insights into toxin dynamics and enabling untargeted metabolic evaluation.

## 1. Introduction

Chronic kidney disease (CKD) is a progressive disorder characterized by a gradual decline in renal function. The global burden of CKD is significant, with its progression influenced by a variety of underlying conditions, including diabetes mellitus, hypertension, and primary glomerulonephritis [1]. As kidney function deteriorates, the kidneys’ ability to filter and excrete metabolic products diminishes, resulting in the accumulation of toxic substances in the blood. These substances, known as uremic toxins, are recognized as key contributors to the complications and comorbidities associated with CKD [2].

Of particular concern are the protein-bound uremic toxins indoxyl sulfate (IndS) and *p*-cresyl sulfate (pCS), which have been linked to the exacerbation of CKD-related complications [3]. These toxins are derived from the microbiota-mediated metabolism of amino acids in the gut and are absorbed into the bloodstream, where they bind to plasma proteins, particularly albumin. Their accumulation is particularly problematic in CKD, as these compounds are poorly removed by hemodialysis, contributing to the persistence of toxicity in patients with advanced kidney disease. In particular, IndS and pCS promote inflammation, oxidative stress, and endothelial dysfunction, and their retention is closely associated with cardiovascular morbidity, as well as the progression of renal fibrosis [4]. Elevated levels of IS and pCS in the blood are considered not only markers of kidney dysfunction but also predictors of adverse clinical outcomes, including increased mortality [4]. Furthermore, these toxins may alter the pharmacokinetics of certain drugs by modifying their protein binding, further complicating the management of CKD patients [5].

The accurate quantification of uremic toxins is crucial for understanding their role in CKD progression and for developing strategies to mitigate their harmful effects. Traditional methods for measuring uremic toxins in serum, such as capillary electrophoresis [6,7,8], are often limited by low sensitivity and specificity. Recently, liquid chromatography coupled with tandem mass spectrometry (LC–MS/MS) has emerged as a powerful analytical technique for the precise and reliable quantification of uremic toxins. Thus, LC–MS/MS offers several advantages, including high sensitivity and specificity, selectivity, accurate quantification, structural information, molecular identification, the ability to simultaneously quantify multiple compounds in a single sample, and comprehensive data acquisition [9,10,11]. On their site, liquid chromatography–high-resolution mass spectrometry (LC–HRMS) has shown sensitivity and quantitative performance comparable to those of LC–MS/MS for the measurement of metabolites [12]. Additionally, HRMS provides more accurate masses than triple-quadrupole instruments with lower resolution [12]. Furthermore, high-resolution full-scan acquisitions allow for both simultaneous quantitative analysis and untargeted compound identification [13]. These factors, along with good versatility and increasing affordability, explain the growing interest in LC–HRMS-based analytical methods in clinical and research laboratories. In this context, the aim of the present study was to validate a straightforward, routine LC–HRMS method for quantifying IndS and pCS in human serum, while also enabling untargeted compound identification within the same analysis.

## 2. Results and Discussion

### 2.1. Characteristics of the Method

For the quantitative measurement of IndS and pCS, protein precipitation was performed on 50 µL of serum sample using 340 µL of methanol. We used IndS-13C_6_ and pCS-d_7_ as internal standards (ISs), based on the commercial availability of isotopically labelled uremic toxins. Mobile phases were methanol (0.1% formic acid) and water (0.1% formic acid). Both methanol and acetonitrile with formic acid have been previously used as organic solvents in mobile phase in other LC–MS methods (Table 1). Additionally, the sample volume and the measurement range are also consistent with previously published methods. The LLOQ for both metabolites is 100 ng/mL, which is consistent with previous studies, where the LLOQ range typically falls between 1 and 500 ng/mL. Table 1 presents the performance characteristics of previously validated methods and our method for IndS and pCS quantification in serum. The main differences between our method and other previously used methods include the use of micro-LC and HRMS. To the best of our knowledge, our method is the first validated method for IndS and pCS measurement in serum taking advantage of any of these characteristics. The stationary phase was a micro-LC HALO 90 Å C18 (100 × 0.3 mm, 2.7 µm; Advanced Materials Technology, Wilmington, DE, USA), with an inner diameter of only 0.3 mm. The use of micro-LC allowed a flow rate of only 10 µL/min, which resulted in a very low consumption of mobile phase per sample (0.1 mL). Also, in contrast to previously validated methods, the current method takes advantage of HRM acquisition and uses the exact mass (deprotonated) of each analyte for quantification. Additionally, full scan allows us to obtain the full mass spectrum of the analytes, with the added possibility of untargeted compound identification. The Supplemental Materials Section summarizes the protocol and main acquisition settings and includes a serum sample, LLOQ, and blank chromatogram.

### 2.2. Method Validation

#### 2.2.1. Linearity of the Calibration Curves

Calibration curves were created by diluting the working solution in water to prevent potential bias from varying levels of endogenous IndS and pCS in serum. The LC–HRMS method demonstrated linearity for both uremic toxins, achieving r^2^ values greater than 0.99. The calibration samples demonstrated an accuracy ranging from 97% to 105% for IndS and 86% to 104% for pCS, with relative standard deviations (RSDs) < 15% (Table 2).

#### 2.2.2. Matrix Effect

**Figure 1:**
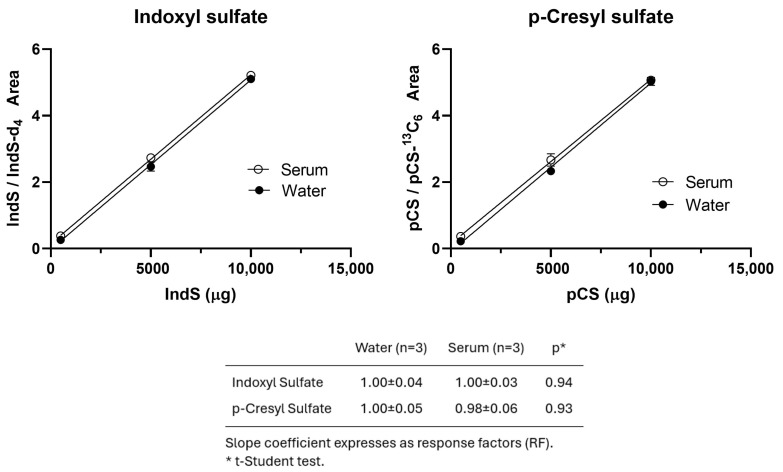
Indoxyl sulfate (IndS) and *p*-Cresyl sulfate (pCS) spiked curves in human serum compared with the respective curves in water.

Additional validation procedures were included to assess the suitability of calibrations prepared in water to quantify serum samples. Figure 1 shows the comparison of the IndS and pCS recoveries spiked in serum samples or in water. No significant differences were observed between the slope coefficients (α) of the curves constructed in aqueous solutions and those spiked in serum. The response factors (RFs) were calculated as α_spiked_serum_/α_water_. Utilizing RFs for the serum-spiked samples did not improve accuracy or precision, sustaining a parsimonious approach that does not necessitate compensation for different matrices.

#### 2.2.3. Accuracy and Imprecision

The inter-assay accuracy and imprecision values of three quality control (QC) levels, as well as the inter-assay imprecision values for a serum sample also met the validation requirements (<15%) and are summarized in Table 3. The lowest calibrator (100 ng/mL) was selected as the lower limit of quantification (LLOQ), and its accuracy and imprecision also fulfilled the validation criterion (<20%) for both uremic toxins. In addition, accuracy was also evaluated in serum samples by spiking IndS and pCS. The LC–HRMS method demonstrated accuracies ranging from 92 to 109%, based on three replicate measurements of serum samples spiked with 5000 and 10,000 ng/mL of each metabolite.

#### 2.2.4. Recovery, Selectivity, and Carry-over

Recoveries evaluated by comparing the areas of IndS-^13^C_6_ and pCS-d_7_ in non-extracted methanolic solutions with those in extracted serum samples ranged from 74 to 91%. Analysis of 50 different human serum samples did not reveal additional interfering signals for the uremic toxins and the internal standards (ISs). Among these samples, similar ion ratios were observed between the quantifying ions (*m*/*z* 212.0023 for IndS and *m*/*z* 187.0071 for pCS) and each of the two qualifying ions (*m*/*z* 107.0502 and *m*/*z* 79.9574 for IndS; *m*/*z* 132.0455 and *m*/*z* 79.9574 for pCS) from the MS/MS scans. Finally, there were no carry-over effects observed after injecting blank samples following the injection of a standard with 100,000 ng/mL, as well as serum samples up to 70,000 ng/mL of IndS and pCS.

#### 2.2.5. Stability of Serum Samples and Stability of the Extracts on the Autosampler

The stability of IndS and pCS in serum was evaluated in two samples stored at 4 °C (24 h), stored at room temperature (24 h), and after three freeze and thaw cycles. The accuracy results of two different serum samples ranged from 89 to 117% in all these three different conditions (Table 4). These results agree with those reported by Lin et al., who found that both analytes remained stable at 4 °C for 7 days and at −20 °C and −70 °C for 3 months [15]. On the other hand, the extracts were stable for up to 48 h in the carousel inside the autosampler (8 °C). The accuracy for extracted QC and serum samples ranged from 93% to 107% up to 48 h for both analytes (Table 5).

### 2.3. Method Application

#### 2.3.1. IndS and pCS Serum Levels Measurements Correlate with Renal Function

**Figure 2:**
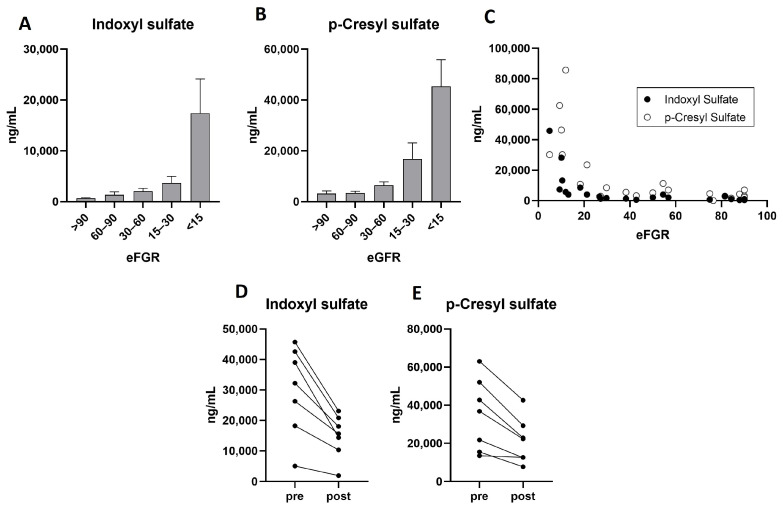
(**A**,**B**) Application of the method to measure serum indoxyl sulfate (**A**) and *p*-cresyl sulfate (**B**) in patients with varying estimated glomerular filtrations rates (eGFRs). (**C**) Correlation between serum levels of uremic toxins and eGFRs. (**D**,**E**). Application of the method to measure serum levels of indoxyl sulfate (**D**) and *p*-Cresyl sulfate (**E**) before and after hemodiafiltration (*n* = 7).

Figure 2A,B show the serum levels of IndS and pCS, respectively, in samples from patients with different estimated glomerular filtration rates (eGFRs). Specifically, patients were classified into five groups: eGFR > 90, eGFR 60–90, eGFR 30–60, eGFR 15–30, and eGFR < 15. IndS levels (ng/mL) were, respectively, 678 ± 114, 1339 ± 603, 2074 ± 573, 3661 ± 1320, and 17,423 ± 6731 (*p* < 0.05). pCS levels (ng/mL) were, respectively, 3223 ± 1031, 3403 ± 701, 6482 ± 1350, 16,803 ± 623, and 45,327 ± 1059 (*p* < 0.01). Results indicate that both uremic toxins significantly increased in serum with the worsening of the renal function, as previously described [2,3]. As can be observed in Figure 2C, the increase in serum toxins is particularly pronounced at very low glomerular filtration rates, which is consistent with previous studies [15,16,17,18,19,20,21]. Of note is that the accumulation of both uremic toxins further contributes to chronic kidney disease (CKD) progression through oxidative stress, renal fibrosis, and inflammation, while promoting cardiovascular disease (CVD) by enhancing vascular inflammation, calcification, and atherogenesis [19,20].

#### 2.3.2. IndS and pCS Serum Levels Measurements Correlate with Renal Function

The quantification of IndS and pCS in serum is also valuable for evaluating the effectiveness of their removal during hemodialysis [22,23,24]. To assess the method’s ability to detect differences between serum samples collected before and after hemodialysis, IndS and pCS were quantified in serum samples obtained from seven patients, both prior to and following hemodiafiltration. All patients exhibited notably decreased levels after hemodiafiltration, except for one patient with residual renal function, who showed a similar concentration of pCS before and after the procedure (Figure 2D,E). The mean serum levels of IndS were 29,888 ± 5490 ng/mL before hemodiafiltration and 14,905 ± 7099 ng/mL after, representing a mean decrease of 50 ± 9%. Similarly, the mean serum levels of pCS were 35,073 ± 7175 ng/mL before hemodiafiltration and 21,422 ± 4527 ng/mL after, resulting in a mean decrease of 43 ± 6%. Recently, this method has been used to compare the efficacy of different dialysates in removing both toxins [25].

#### 2.3.3. Simultaneous Untargeted HRMS

To demonstrate the method’s capability for simultaneous quantification of IndS and pCS, as well as for untargeted analysis, a pilot untargeted analysis was conducted on serum samples from seven patients collected before and after hemodiafiltration. This analysis was focused on metabolites with physicochemical characteristics similar to those of IndS and pCS (similar mass and negative polarity ionization). A total of 579 distinct chromatographic peaks were observed. However, statistical analysis was limited to peaks with chromatographic signals at least five times higher than those observed in blank samples that were processed using the same procedure as the serum samples. A total of 479 metabolites met these criteria, and their peak areas before and after hemodiafiltration were compared. Using these stringent criteria, 28 metabolites were found to be differentially underexpressed in serum samples collected after hemodiafiltration. Mass spectra comparison with reference libraries led to the identification of 14 endogenous metabolites that result from various metabolic processes such as amino acid metabolism, purine metabolism, glucose metabolism, and gut microbiota activity (Table 6). Many of them are processed through phase II reactions (e.g., sulfation, glucuronidation, and amino acid conjugation) in the liver and kidneys and are then filtered and excreted by the kidneys. Although the findings are limited by the small number of patients, the results highlight the method’s ability to enable simultaneous untargeted metabolomic analyses.

## 3. Materials and Methods

### 3.1. Chemical Reagents

Indoxyl sulfate potassium salt, *p*-cresyl sulfate and indoxyl sulfate ^13^C_6_ potassium salt were purchased from Merck (Darmstadt, Germany); the *p*-cresyl sulfate-d7 was from Toronto Research Chemicals (North York, ON, Canada). Formic acid and LC–MS grade methanol were obtained from Merck (Darmstadt, Germany). Ultrapure water was produced using a Millipore Milli-Q purification system (Merck, Darmstadt, Germany).

### 3.2. Preparation of Stock Solutions, Working Solutions, Calibrator, and Quality Control Samples

Calibration curves were prepared using aqueous solution of IndS and pCS due to the endogenous presence of both compounds in human serum. The analytical response differences between aqueous solutions and serum were assessed following a recovery assessment. A stock solution of IndS and pCS was prepared at a concentration of 100 mg/mL in water and stored at −20 °C. Working solutions were prepared by mixing and diluting the stock solutions in water to a final concentration of 100,000 ng/mL for each metabolite. Seven-point calibration curves (100, 500, 1000, 2500, 5000, 10,000, and 40,000 ng/mL) were prepared by diluting the working solution in water. For the QC, three levels were prepared (750, 3500, and 30,000 ng/mL). The IS stock solutions of indoxyl sulfate ^13^C_6_ and *p*-cresyl sulfate-d_7_ were prepared at a concentration of 20 µg/mL, and working solutions were prepared at a final concentration of 2 µg/mL for each metabolite.

### 3.3. Sample Preparation

For the quantitative measurements of IndS and p-CS, 25 µL of the each IS working solution were added to 50 µL serum samples, calibrators or QC, and mixed with 340 µL of methanol. After centrifugation at 15,000× *g* for 10 min, the organic phase was transferred to a clean 5 mL glass tube and evaporated to dryness under nitrogen at 37 °C. The residue was reconstituted in 100 µL of water and centrifuged at 15,000× *g* for 5 min, and the supernatant was transferred into an autosampler glass vial. A schematic of the sample preparation process is provided in Supplementary Materials.

### 3.4. Instrumentation

The analysis was performed by LC–HRMS using a Dionex ultiMate3000 RSLC system coupled to a hybrid quadrupole-Orbitrap mass spectrometer (Orbitrap Exploris 120, Thermo Fisher Scientific, Bremen, Germany), equipped with an electrospray ionization source (ESI). The LC column was a HALO 90 Å C18, 2.7 µm, 0.3 × 100 mm (Advanced Materials Technology, Wilmington, DE, USA), which was connected to a Thermo Scientific Pep Map Neo Trap Cartridge Holder. The column oven was maintained at 40 °C, and the mobile phases A and B were, respectively, water (0.1% formic acid) and methanol (0.1% formic acid). One microliter was injected into the LC instrument and the total run time was 10 min. The gradient elution of the NC pump (flow rate of 10 µL/min) began at 0.5% solvent B, gradually increasing to 95% over 6.5 min. The gradient was maintained at 95% B until 7.5 min, after which it returned to the initial condition of 0.5% B by minute 8. From minutes 8 to 10, the gradient was held constant at 0.5% B. A loading pump was connected to the LC column with a flow rate of 100 µL/min from minute 0 to 0.25 and from minute 8 to 10. From minutes 0.25 to 8, the loading pump was not connected to the LC column. The ion source was operated in both negative and positive ion modes using the following settings: positive ion 3400 V, negative ion 4000 V, sheath gas 27, aux gas 10, ion transfer tube temperature 350 °C, and vaporizer temperature 100 °C. The instrument was programmed simultaneously both in full-scan and targeted modes. The full-scan mode was programmed with the following settings: scan range, 120–320 *m*/*z*; resolution, 30,000; RF Lens (%), 70; and polarity, negative. Targeted MS scans were based on deprotonated exact masses for uremic toxin quantification using normalized collision energy, resolution, 15,000, and the MS/MS scan were set at a resolution of 15,000. Targeted *m*/*z* for IndS, IndS-^13^C_6_, pCS, and pCS-d_7_ were, respectively, 212.0023, 218.0224, 187.0071, and 194.0510. Data acquisition and analysis were achieved by using the TraceFinder 5.1. software (Thermo Fisher Scientific, Bremen, Germany).

### 3.5. Method Validation

For the validation of the method, the ICH guideline M10 [26] was followed.

#### 3.5.1. Linearity of the Calibration Curves

For the evaluation of linearity, calibration curves were analyzed on five different days for both analytes across a concentration range of 100 to 40,000 ng/mL, using seven calibration points (100, 500, 1000, 2500, 5000, 10,000, and 40,000 ng/mL). Slope, y-intercept, and correlation coefficient were calculated for each standard curve. A 1/X weighted linear regression was used. A value of r^2^ ≤ 0.99 was required to pass this validation step. The precision and accuracy versus the nominal concentration of the calibrator levels were also calculated. The back-calculated concentrations were acceptable when within ±15% of the nominal values. The LLOQ was set at the lowest calibration standard value (100 ng/mL), and a ±20% accuracy was considered [26].

#### 3.5.2. Matrix Effect

The analytical responses of IndS and pCS were evaluated to confirm that the calibration curves established in aqueous solution standards could be used to quantify serum samples. The slope coefficients (α) of three-point spiked curves in human serum from three different sources were compared with their respective curves in water. Response factors (RFs) were calculated as the ratio α_spiked serum_/α_spiked water_. The concentrations of the serum samples calculated both with and without RF correction were used to determine the sum of the absolute relative residuals as (C_spiked_ − C_nominal_)/C_nominal_.

#### 3.5.3. Accuracy and Imprecision

The method’s accuracy and precision were evaluated by analyzing the back-calculated results from multiple measurements of three QC levels (750, 3500, and 30,000 ng/mL). Additionally, the accuracy and precision at both the lower limit of quantification (LLOQ) and upper limit of quantification (ULOQ) were examined, with the LLOQ set at 100 ng/mL and the ULOQ at 40,000 ng/mL, based on the lowest and highest calibration standards. Inter-day accuracy and precision were determined by performing the analyses over five separate days. For accuracy to be acceptable, the average results needed to fall within 100 ± 15% of the target value, while acceptable imprecision reported as relative standard deviation (%RSD) was defined as <15% [26]. For the LLOQ, accuracy had to fall within 100 ± 20%, with imprecision under 20% [26]. Additionally, accuracy of the spiked IndS and pCS was determined in serum samples by comparing the measured (observed) concentrations with the unspiked concentrations (basal) following the formula, Recovery (%) = (C_observed_ − C_basal_)/C_spiked_.

#### 3.5.4. Recovery, Selectivity, and Carry-over

Recovery was evaluated at three concentrations of internal standards (500, 5000, and 10,000 ng/mL) by comparing the areas of non-extracted methanolic working solutions with those in extracted serum samples containing the same number of internal standards. The selectivity was evaluated by analyzing 50 different human serum samples and was indicated by the lack of endogenous interferences at the retention times of both metabolites and the IS. The carry-over was evaluated by injecting one microliter of water after the injection of the higher standard (ULOQ) on three separate runs.

#### 3.5.5. Stability of Serum Samples and Stability of the Extracts on the Autosampler

The stability of IndS and pCS in serum was evaluated in two different concentration levels by measurements of three replicates of serum samples stored under different conditions and durations: three freeze–thaw cycles, ambient temperature for 24 h and 4 °C for 24 h. The stability of the extracts on the autosampler was evaluated by reinjecting one QC and one serum sample extracts stored inside the autosampler (8 °C) for 24 and 48 h.

### 3.6. Method Application and Statistical Analysis

The concentration levels of IndS and pCS were measured in serum samples from 20 patients with varying degrees of renal function. Patients were classified into four different groups based on their estimated glomerular filtration rate (eGFR) values (mL/min/1.73 m^2^), according to current guidelines for chronic kidney disease [27]. Patients were grouped as follows: eGFR > 90 (*n* = 5), eGFR 60–90 (*n* = 5), eGFR 30–60 (*n* = 5), eGFR 15–30 (*n* = 5), and eGFR < 15 (*n* = 5). The concentration results of the uremic toxins are expressed as mean ± SEM, and *p* values were calculated using one-way ANOVA with Tukey’s multiple comparison test. In addition, the concentration levels of IndS and pCS were quantified in serum samples from seven patients before and after hemodiafiltration. The concentration results are expressed as mean ± SEM, and *p* values were calculated using paired *t*-test.

Alongside the validation of the quantification for these two uremic toxins, a simultaneous untargeted analysis was conducted with the aim of identifying other potentially toxic metabolites that decrease after hemodiafiltration. To achieve this, the method was configured such that the mass spectrometer detector performed a full-scan analysis (mass range of 120–320) concurrently with the SIM analysis. The obtained data were then analyzed using the software Compound Discoverer v. 3.3.0.550 (Thermo Fisher Scientific). Libraries included for the identification of metabolites were mzCloud (Endogenous Metabolites and Natural Toxins), Bamba’s lab 598 polar metabolites (Kyushu University), and HMDB from mzVault. A comparison was made between serum samples from patients (*n* = 7) collected before and after the hemodialysis procedure. Differences were considered statistically significant for *p* values (*t*-test) below 0.05.

Statistical analyses were conducted using GraphPad Prism 6 (GraphPad Prism Software Inc., San Diego, CA, USA) and RStudio Team (2020), which provides an integrated development environment for R. This study adhered to the ethical guidelines outlined in the Declaration of Helsinki established by the World Medical Association. This study was performed in agreement with the criteria of the Investigation and Ethics Committee of the Hospital Clinic (Barcelona, Spain).

## 4. Conclusions

An LC–HRMS method was validated for the quantitative measurement of indoxyl sulfate and *p*-cresyl sulfate in human serum, which is helpful for monitoring the accumulation of these uremic toxins and their removal by hemodiafiltration. In addition, full-scan acquisition allows for simultaneous untargeted high-resolution compound detection.

## Figures and Tables

**Table 1:** Characteristics of LC–MS-validated methods for serum indoxyl sulfate (IndS) and *p*-cresyl sulfate (pCS) quantification.

Ref.	Sample Volume	Internal Standards	Sample Preparation	Calibration Range (ng/mL)	LLOQ (ng/mL)	Mobile Phase	Stationary Phase	MS	RT (min)	Quantification Ions
[14]	50 µL	IndS-d_4_ pCS-d_7_	Methanol	1–50,000	1	H_2_O + 0.1% FA ACN1	HPLC Accucore PFP column (100 × 2.1 mm, 2.6 μm)	MS/MS	IndS: 5.8 pCS: 6.2	IndS: 212.1 > 80.0 pCS: 187.1 > 107.1
[15]	50 µL	IndS-^13^C_6_ pCS-d_4_	ACN	50–50,000	50	H_2_0 + 0.1% FA ACN1 + 0.1% FA	UPLC Acquity UPLC BEHC 18 column (100 × 2.1 mm, 1.7 mm)	MS/MS	IndS: 1.1 pCS: 1.6	IndS: 212.04 > 80.14 pCS: 186.98 > 107.03
[16]	50 µL	IndS-d_4_ pCS-d_7_	ACN	100–10,000	100	H_2_O + 0.1% FA ACN1 + 0.1% FA	HPLC Ultra PFP Propyl column (50 × 2.1 mm, 5 μm)	MS/MS	IndS: 0.5 pCS: 0.6	IndS: 211.9 > 79.9 pCS: 186.8 > 106.8
[17]	50 µL	IndS-d_4_ pCS-d_7_	Methanol	200–80,000 250–80,000	IndS: 200 pCS: 250	10 mM ammonium formate (pH 4.3) ACN1 + 0.1% FA	UPLC Acquity BEH C18 (100 × 2.1 mm, 1.7 μm)	MS/MS	IndS: 2.1 pCS: 3.3	IndS: 212.0 > 80.4 pCS: 186.9 > 107.5
[18]	10 µL	IndS-d_4_ pCS-d_4_	ACN + 0.1% FA	485–50,000 534–26,324	IndS: 485 pCS: 534	Methanol/H_2_O (20:80, *v*/*v*) + 0.1% acetic acid Methanol/H_2_O (80:20, *v*/*v*) + 10 mmol/L ammonium acetate	HPLC Scherzo SS-C18 (50 mm × 2 mm, 3 μm)	MS/MS	IndS: 12.7 pCS: 11.6	IndS: 212.1 > 131.9 pCS: 186.8 > 106.9
OUR	50 µL	IndS-^13^C_6_ pCS-d_7_	Methanol	100–40,000 100–40,000	100	H_2_O + 0.1% FA Methanol + 0.1% FA	micro-LC HALO 90 Å C18 (100 × 0.3 mm, 2.7 µm)	HRMS	IndS: 2.6 pCS: 3.1	IndS: 212.0023 pCS: 187.0071

MS: Mass Spectrometry; RT: Retention time; ACN: acetonitrile; FA: formic acid, HPLC: High-Performance Liquid Chromatography, HRMS: High-Resolution Mass Spectrometry, LC: Liquid Chromatography, MS/MS: Tandem Mass Spectrometry, UPLC: Ultra-Performance Liquid Chromatography.

**Table 2:** Inter-day accuracy and imprecision values of the calibration curve standards (*n* = 5 independent calibration runs).

ng/mL	Indoxyl Sulfate		*p*-Cresyl Sulfate	
	Accuracy (%)	Imprecision (%)	Accuracy (%)	Imprecision (%)
100	105	11.7	100	15.1
500	100	8.8	100	6.8
1000	97	5.4	99	11.4
2500	97	9.0	86	14.0
5000	97	8.6	95	8.4
10,000	102	5.3	104	5.8
40,000	101	4.7	97	5.5

**Table 3:** Inter-day accuracy and imprecision values of quality controls (QCs) and inter-day imprecision values of a serum sample (*n* = 5).

	Concentration (ng/mL)	Accuracy (%)	Imprecision (%)
Indoxyl sulfate			
LLOQ	100	105	11.7
QC	750	100	8.2
QC	3500	92	8.8
QC	30,000	101	0.7
Serum	1978	-	3.2
*p*-Cresyl sulfate			
LLOQ	100	100	15.1
QC	750	96	5.8
QC	3500	87	12.6
QC	30,000	99	1.2
Serum	8439	-	11.8

**Table 4:** Stability of IndS and pCS in human serum expressed as accuracy.

	Concentration	Accuracy (%)		
	ng/mL	24 h (25 °C)	24h (4 °C)	F&T
Indoxyl sulfate	3956	95	102	95
	7597	114	116	117
*p*-Cresyl sulfate	19,627	98	98	105
	31,248	89	98	101

F&T: three freeze-thaw cycles.

**Table 5:** Stability of IndS and pCS in the extracts stored on the autosampler (8 °C).

	Concentration		Accuracy (%)
	(ng/mL)	24 h	48 h
Indoxyl sulfate			
QC	3500	106	93
Serum	6831	102	96
*p*-Cresyl sulfate			
QC	3500	106	107
Serum	37,674	103	102

**Table 6:** List of metabolites with physicochemical characteristics similar to IndS and pCS, identified as the most underexpressed after hemodiafiltration in a simultaneous untargeted analysis alongside the quantification of IndS and pCS.

Name	Molecular Formula	*m*/*z*	Log 2 Fold Change	*p*	Brief Summary
3-(Sulfooxy)benzenepropanoic acid	C_9_H_10_O_6_S	245.01958	−5.14	5.6 × 10^−4^	Sulfonated metabolite of a phenolic compound
(Carbamoylamino)(4-hydroxyphenyl)acetic acid	C_9_H_10_N_2_O_4_	209.06393	−4.26	1.3 × 10^−2^	Conjugated metabolite from amino acid metabolism
1,3-Dimethyluric acid	C_7_H₈N_4_O_3_	195.05220	−3.13	4.4 × 10^−2^	Breakdown product of purine metabolism
Gluconic acid	C_6_H_12_O_7_	135.02982	−3.11	4.0 × 10^−3^	Glucose metabolism
Uric Acid	C_5_H_4_N_4_O_3_	167.02087	−2.80	1.2 × 10^−3^	Purine metabolism
Perseitol	C_7_H_16_O_7_	152.06112	−2.78	5.8 × 10^−3^	Sugar alcohol metabolite
L-α-Aspartyl-L-phenylalanine	C_13_H_16_N_2_O_5_	279.10598	−2.73	1.6 × 10^−4^	Protein metabolism
4-phenolsulfonic acid	C_6_H_6_O_4_S	172.99871	−2.59	5.2 × 10^−2^	Metabolite of phenolic compounds
2-Hydroxyhippuric acid	C_9_H_9_NO_4_	194.04576	−2.44	1.1 × 10^−2^	Metabolite of aromatic compounds
Phenylac-gln-OH	C_13_H_16_N_2_O_4_	263.10347	−2.36	2.9 × 10^−4^	Amino acid metabolism
*p*-Cresyl glucuronide	C_13_H_16_O_7_	283.08240	−2.23	6.0 × 10^−3^	*p*-Cresyl conjugated with glucuronic acid
Hippuric acid	C_9_H_9_NO_3_	178.05065	−2.12	8.4 × 10^−3^	Metabolism of benzoic acid
Homovanillic acid	C_9_H_10_O_4_	181.05065	−1.82	2.4 × 10^−2^	Metabolite of dopamine, a neurotransmitter
Erythritol	C_4_H_10_O_4_	121.05046	−1.26	3.3 × 10^−2^	Sugar alcohol

## Data Availability

The original contributions presented in this study are included in this article/Supplementary Materials. Further inquiries can be directed to the corresponding authors.

## Supplementary Materials

The following supporting information can be downloaded at: https://www.mdpi.com/article/10.3390/molecules30040782/s1, Figure S1: Schema of the assay procedure for the determination of IndS and pCS in human serum samples; Figure S2: Chromatogram of a serum sample (A), LLOQ (B), and blank (C) measured by the LC–HRMS method. Table S1: Retention times and monitored ions of the uremic toxins and internal standards.
