# Supplementary material for: Validation of an LC–HRMS Method for Quantifying Indoxyl Sulfate and p-Cresyl Sulfate in Human Serum
Source: Molecules. 2025 Feb 8;30(4):782. doi: 10.3390/molecules30040782 (PMC11857974; doi:10.3390/molecules30040782)
Supplement: Supplementary file 1 [file molecules-30-00782-s001.zip › molecules-3418408-supplementary.pdf]

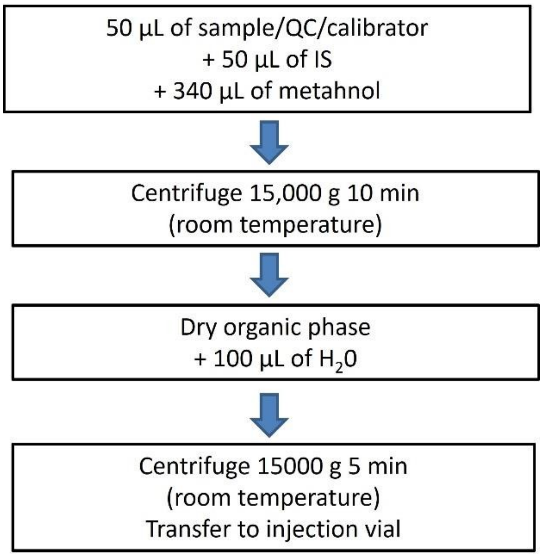

Figure S1. Schema of the assay procedure for the determination of IndS and pCS in human serum samples. IS: internal standard.

A

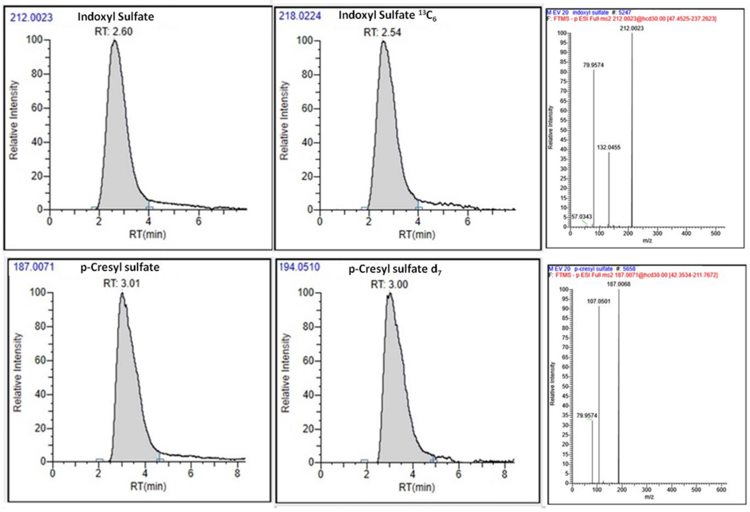

B

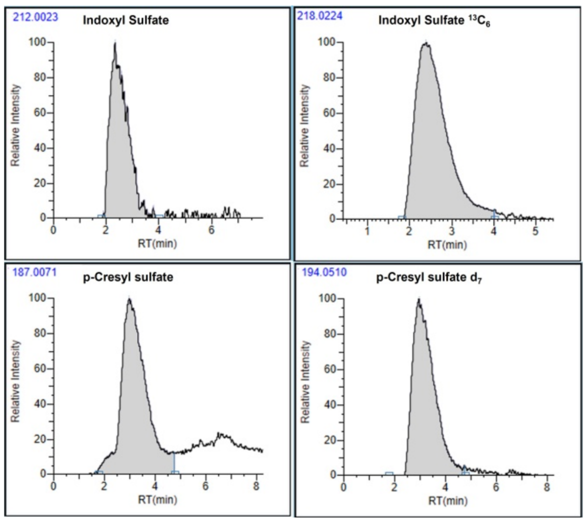

C

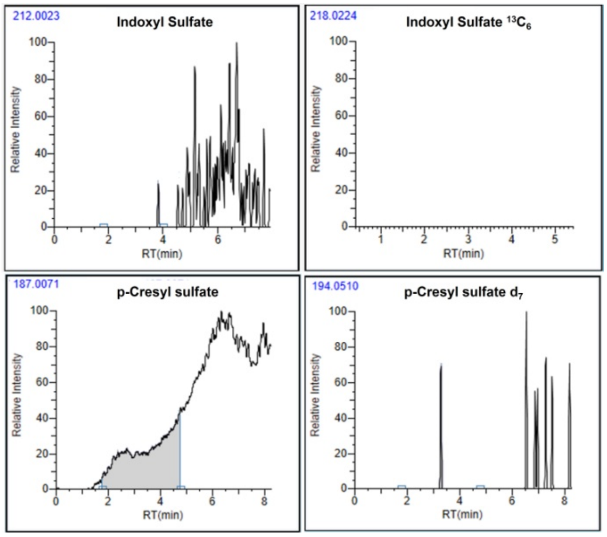

Figure S2. Chromatogram of a serum sample (A), LLOQ (B) and blank (C) measured by the LC-HRMS method

Table S1. Retention times and monitored ions of the uremic toxins and internal standards.

Compound	Formula	Target ion	m/z	Ret. Time (min)
IndS	$C_8H_7NO_4S$	$[M\text{-}H]^-$	212.0023	2.60
IndS-$^{13}C_6$	$^{13}C_6C_2H_7NO_4S$	$[M\text{-}H]^-$	218.0224	2.54
pCS	$C_7H_8O_4S$	$[M\text{-}H]^-$	187.0071	3.01
pCS-d_7	$C_7HD_7O_4S$	$[M\text{-}H]^-$	194.0510	3.00
